# Fever, Syncope, and the Brugada Dilemma: Navigating the Complexities of ICD Decision-Making in an Atypical Presentation

**DOI:** 10.1155/cric/5536305

**Published:** 2025-10-24

**Authors:** Lorna Devkota, Michael Hoffer-Hawlik, Felix Yang, Albert S. Jung

**Affiliations:** ^1^Department of Medicine, New York University Grossman School of Medicine, New York, New York, USA; ^2^Department of Cardiology, New York University Grossman School of Medicine, New York, New York, USA

## Abstract

Brugada syndrome (BrS) is a rare inherited cardiac channelopathy associated with ventricular arrhythmias and sudden cardiac death (SCD), often in individuals with structurally normal hearts. It is diagnosed by a Type 1 electrocardiographic (EKG) pattern—coved ST-segment elevation in the right precordial leads. Fever is a known trigger that can unmask Brugada patterns by worsening sodium channel dysfunction. We present the case of a 20-year-old male with a fever and an episode of syncope prior to admission who had an EKG showing a Type 1 Brugada pattern. Procainamide challenge was negative, but an atypical right bundle branch block suggested sodium channel dysfunction. Despite the absence of structural heart disease, an implantable cardioverter defibrillator (ICD) was placed for primary prevention of SCD. This case underscores the diagnostic and management challenges in intermediate-risk BrS patients.

## 1. Introduction

Brugada syndrome (BrS) increases the risk of ventricular arrhythmias (VAs) and sudden cardiac death (SCD) [1]. It is characterized by a Type 1 electrocardiographic (EKG) pattern—coved ST-segment elevation ≥ 2 mm followed by a negative T-wave in the right precordial leads (V1–V3) [2]. BrS is most commonly associated with pathogenic variants in cardiac sodium channels, particularly the *SCN5A* gene [3].

Risk stratification in BrS typically considers spontaneous Type 1 EKG pattern, history of syncope or cardiac arrest, family history of SCD, inducibility of arrhythmias during electrophysiologic studies, and presence of genetic variants [1]. Tools such as the Shanghai Score System and other risk scores have been developed to estimate BrS likelihood and guide ICD implantation [4]. However, these tools are often less predictive in patients with intermediate risk, such as those with fever-induced Brugada patterns or nonclassical symptoms, where arrhythmic risk is uncertain and management decisions are more complex [5].

Fever is a recognized trigger that can unmask Brugada EKG patterns and heighten arrhythmic risk by further impairing sodium channel function [6]. These transient findings complicate diagnostic certainty and therapeutic planning, particularly when considering ICD placement for primary prevention of SCD. We report a case of BrS in a young patient with fever and syncope presenting with intermediate risk of SCD.

## 2. Case Description

**Figure 1:**
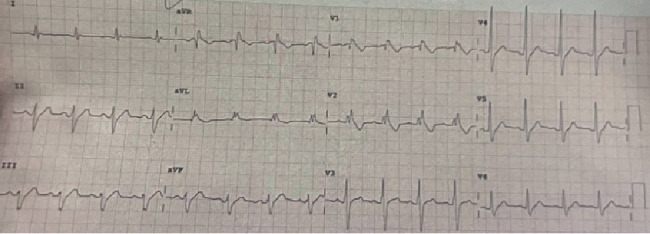
EKG on admission demonstrated characteristic Type 1 coved-shaped ST-segment elevation in Lead V1.

A 20-year-old male with no prior medical history presented with a fever of 103°F and a syncopal episode while ascending stairs. Witness accounts were unclear regarding complete loss of consciousness. Per family accounts, the patient may have briefly lost consciousness during the fall at home but quickly regained consciousness, after which he was alert and oriented. The patient did not recall falling or hitting his head. No seizure-like activity (e.g., eye-rolling, tongue biting, or incontinence) was reported. He denied taking any medications, and there was no reported relevant family history. No previous episodes of syncope were reported. Emergency medical services were contacted, and the patient was brought to an outside hospital where an EKG revealed a Brugada Type 1 pattern (Figure 1). On admission, vital signs and physical examination were unremarkable. Echocardiography demonstrated a normal left ventricular ejection fraction, and cardiac MRI showed a mildly reduced ejection fraction of 45% with no late gadolinium enhancement. No pertinent lab values were noted. Computed tomography (CT) head and chest radiograph were unremarkable.

**Figure 2:**
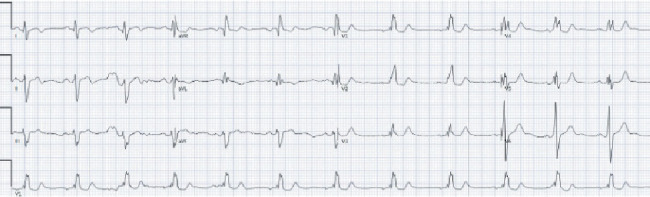
Baseline EKG demonstrated a right bundle branch block (RBBB).

**Figure 3:**
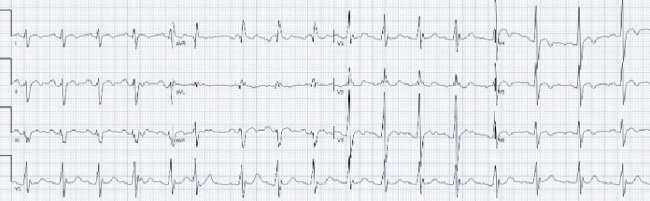
EKG during procainamide challenging demonstrated dynamic changes in the terminal portion of QRS complexes in Leads V1–V3.

Electrophysiology (EP) service was consulted for consideration of subcutaneous implantable cardioverter defibrillator (S-ICD) placement. Given the patient's syncopal episode and Brugada pattern demonstrated on EKG, an electrophysiological study (EPS) with procainamide challenge was recommended. Procainamide was administered at 1000 mg intravenous infusion over 20 min with continuous telemetry monitoring and serial EKGs in high-intercostal space lead position. The patient's baseline EKG demonstrated a right bundle branch block (RBBB) (Figure 2). During the procainamide challenge, an atypical RBBB was observed without diagnostic ST segment elevation, thus demonstrating a negative study for Type 1 Brugada pattern. However, there were dynamic changes in the terminal portion of the QRS complex with procainamide, suggestive of a sodium channel dysfunction (Figure 3). Findings of the study were discussed with the patient and the family. While the procainamide challenge did not reveal a Type 1 Brugada pattern, the EP service believed that BrS may have been unmasked in the context of the patient's fever and syncopal event. After multidisciplinary discussions and shared decision-making with the patient and family, an S-ICD was placed for primary prevention of SCD.

## 3. Discussion

BrS is a genetic ion channelopathy associated with an increased risk of syncope and SCD due to VAs [7]. BrS is linked to mutations affecting sodium, potassium, and calcium channels, which can alter transmembrane protein function and channel trafficking [8]. These mutations predispose patients to defective depolarizations and preexcitation that can lead to life-threatening arrhythmias. Mutations causing loss of function in sodium channels—particularly in the SCN5A gene—are sensitive to temperature changes, which can influence action potential duration [9, 10].

Although BrS is diagnosed with a Type 1 EKG pattern without an obvious trigger, there are currently limitations in the diagnostic approach for BrS if the Type 1 pattern is fever or pharmacologically induced. Fever has been shown to not only unmask Brugada-type EKG findings but also increase the risk of ventricular fibrillation or SCD [11]. Elevated temperatures can impair current amplitude and raise the activation voltage of the sodium channel, thereby increasing the risk of reentry and a subsequent arrhythmogenic event [12].

There are several risk stratification scores for BrS that incorporate factors such as syncope, spontaneous EKG changes, and response to programmed electrical stimulation in patients without a previous cardiac arrest [13]. However, these scoring systems are often of limited utility in intermediate risk or fever-induced Type 1 pattern, where the decision to place an ICD for primary prevention is complex [14, 15]. Elucidating the mechanism of syncope in BrS requires differentiation between reflex and arrhythmogenic syncope and informs the subsequent diagnostic workflow [16]. Although syncope in BrS is often regarded as a marker of increased risk for VAs, true arrhythmic syncope is extremely rare, whereas reflex-mediated syncope occurs at rates similar to those in the general population. In one review, the rarity of true arrhythmic syncope was emphasized relative to the prevalence of reflex events [17]. Supporting data from a clinical cohort study demonstrated that more than half of BrS patients presenting with syncope had nonarrhythmic episodes, often preceded by prodromal symptoms or identifiable triggers. These patients demonstrated little to no risk of arrhythmic recurrence during long-term follow-up [18]. When reflex syncope is suspected, tilt table testing or evaluation for alternative etiologies such as orthostatic hypotension, atrioventricular block, or supraventricular tachyarrhythmias should be considered before proceeding to EPS for BrS risk stratification. Exercise testing has also gained interest in BrS. While historically discouraged, recent data indicate that moderate exercise is not universally contraindicated and may provide additional diagnostic value, particularly in patients with exertional symptoms or equivocal ECG findings [19]. Under appropriate supervision, exercise testing has been shown to be safe and may unmask arrhythmic risk or dynamic ECG changes that refine risk stratification. For patients whose syncope is unlikely to be reflex in origin, further stratification should incorporate risk factors such as family history of SCD, concomitant conduction disease, and the presence of a spontaneous Type 1 ECG pattern in conjunction with EPS [20].

Implantation of an ICD in BrS is informed through guideline-directed risk stratification. The 2015 ESC Guidelines designate a Class I indication for ICD implantation in patients with a spontaneous Type 1 EKG pattern and a history of arrhythmic syncope, and a Class IIa indication is designated in those with an inducible Brugada pattern and unexplained syncope [4]. Although our patient's Brugada pattern appeared during a febrile episode, the clinical context raised suspicion for a potential arrhythmic syncopal event. While the procainamide challenge did not reproduce a diagnostic Type 1 pattern, its sensitivity (80%–88%) and specificity (80–90%) do not exclude BrS in high-risk settings [21]. Further, EPS has not been shown to significantly determine differences in the incidence rate of arrhythmogenic events in drug-induced Type 1 BrS patients, thus highlighting the challenge of prognostic risk stratification in this population [22].

This patient's presentation with syncope during a febrile illness and a Brugada Type 1 pattern placed him in the intermediate-risk category, a subpopulation with uncertain clinical risk. Though syncope was not definitively linked to an arrhythmia in this case (due to the absence of rhythm monitoring at the time of the event), the presence of EKG abnormalities—including an atypical RBBB and QRS changes during procainamide challenge—suggested an underlying sodium channelopathy. In such indeterminate cases, long-term monitoring with an implantable loop recorder (ILR) can provide incremental diagnostic yield. The recent BRULOOP study demonstrated that ILRs effectively capture spontaneous arrhythmic events, enabling more accurate classification of syncope and helping to avoid unnecessary ICD implantation in patients who remain at low arrhythmic risk [23]. Further, retrospective studies have shown that children and adolescents are particularly susceptible to fever-induced Brugada patterns and arrhythmic events [24].

The decision regarding the placement of an ICD for primary prevention in young and intermediate-risk BrS patients must factor in the absolute risk of inappropriate shocks or other device complications over a lifetime compared to the risk of SCD development. Genetic mutations associated with BrS, particularly in *SCN5A*, also increase the risk of overlap conduction disease, including sick sinus syndrome, AV block, and long QT syndrome [25]. Thus, ICD device selection is an important consideration to minimize the burden of inappropriate therapies and hardware-related complications. Russo et al. reported no significant differences in inappropriate ICD therapies, device-related complications, or infections between S-ICD and transvenous ICD (TV-ICD) in a propensity-matched study of drug-induced Type 1 BrS patients, although S-ICD was associated with a lower incidence of lead-related device complications [26]. Similarly, Watanabe et al. found no significant difference in inappropriate shock rates between S-ICD and TV-ICD groups in a retrospective study of BrS patients [27]. Taken together, these findings suggest that although both devices provide comparable arrhythmic protection, S-ICD may offer the advantage of fewer lead-related complications and should be considered in device selection.

While the syncope reported in this case could not be definitively attributed to an arrhythmic cause given the lack of EKG monitoring at the time of the event, the combination of clinical and diagnostic findings in the setting of the patient's symptoms ultimately prompted S-ICD placement for primary prevention of SCD. Future studies incorporating additional noninvasive approaches for risk stratification, such as polygenic sequencing across the proband and family or the use of multimodal imaging, may allow for refinement of risk determination of SCD and could inform shared decision-making regarding ICD placement in these indeterminate risk patients [28, 29].

## Data Availability

Research data are not shared.
